# Optimization of the Laser Properties of Polymer Films Doped with *N,N´*-Bis(3-methylphenyl)-*N,N´-*diphenylbenzidine

**DOI:** 10.3390/ma2031288

**Published:** 2009-09-10

**Authors:** Eva M. Calzado, Pedro G. Boj, María A. Díaz-García

**Affiliations:** 1 Dpto. Física, Ingeniería de Sistemas y Teoría de la Señal and Instituto Universitario de Materiales de Alicante, Universidad de Alicante, Alicante-03080, Spain; E-Mail: evace@ua.es (E.M.C.); 2 Dpto. Óptica and Instituto Universitario de Materiales de Alicante, Universidad de Alicante, Alicante-03080, Spain; E-Mail: p.boj@ua.es (P.G.B.); 3 Dpto. Física Aplicada, Unidad asociada UA-CSIC and Instituto Universitario de Materiales de Alicante, Universidad de Alicante, Alicante-03080, Spain

**Keywords:** *N,N´*-bis(3-methylphenyl)-*N,N´*-diphenylbenzidine system (TPD), lasers, amplified spontaneous emission

## Abstract

This review compiles the work performed in the field of organic solid-state lasers with the hole-transporting organic molecule *N,N´*-bis(3-methylphenyl)-*N,N´*-diphenyl-benzidine system (TPD), in view of improving active laser material properties. The optimization of the amplified spontaneous emission characteristics, *i.e.,* threshold, linewidth, emission wavelength and photostability, of polystyrene films doped with TPD in waveguide configuration has been achieved by investigating the influence of several materials parameters such as film thickness and TPD concentration. In addition, the influence in the emission properties of the inclusion of a second-order distributed feedback grating in the substrate is discussed.

## 1. Introduction

Since the discovery in 1996 of stimulated emission in semiconducting polymer films [1,2,3], extensive research has been devoted to the development of solid-state lasers based on semiconducting materials, including not only polymers, but also small organic molecules, oligomers and dendrimers [4,5,6]. A very unique property of organic materials is that, due to their broad photoluminescence (PL) spectrum, the laser wavelength can be tuned over a wide range [7]. Among the various types of organic materials, those that are semiconducting open the possibility of electrical pump. Moreover, those that are soluble, have the additional advantage of easy processability, so they can be deposited in the form of thin films by inexpensive techniques. One of the main challenges in the search of new active laser materials is the achievement of laser thresholds low enough to allow pumping with compact and inexpensive optical sources. Thus, some lasers excited with microlasers the size of a match box [8] or even diode lasers [9,10] have recently been demonstrated.

The simplest method to evaluate the potential of a certain material for use as an active laser medium consists of photopumping films of the material with a stripe of light and identifying the presence of amplified spontaneous emission (ASE). If ASE occurs, a collapse of the width of their PL spectra at certain pump intensity should be observed [2,4,11]. In these cases, the emitted intensity depends on the length of the stripe and follows the equation:

(1) $$I(\lambda )=⎣A(\lambda )I_{p}/g(\lambda )⎦\;\left\{\exp\left[g(\lambda )l\right]-1\right\}$$

where *A(λ)* is a constant related to the cross section for spontaneous emission, *I*_p_ is the pump intensity, *g(λ)* is the net gain coefficient and *l* is the length of the pump stripe [11].

**Figure 1:**
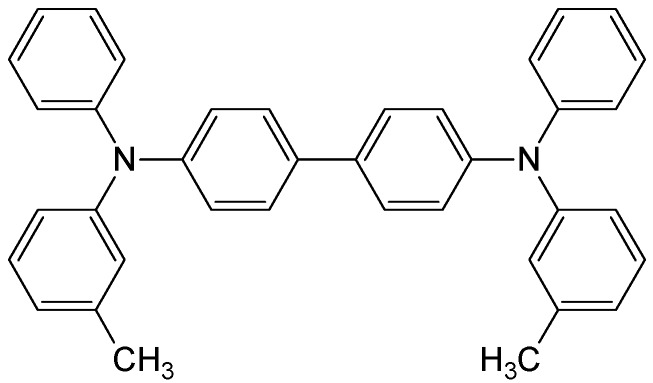
Chemical structure of N,N’-bis(3-methylphenyl)-N,N’-dyphenylbenzidine (TPD).

Concerning molecular semiconducting materials, in recent years numerous molecules and oligomers deposited as thin films have been studied [6,12,13,14,15,16,17]. In many cases, these materials have shown ASE or laser thresholds much lower than those of traditional dyes [18] and comparable to those of various semiconducting polymers [4,6]. The most widely used technique for film preparation has been spin-coating. Typically, solutions containing the active molecule and an inert polymer are deposited over transparent substrates, such as glass or quartz. The main reason for the generally lower ASE thresholds observed with semiconducting materials with respect to dyes, is mostly based on the fact that the former ones can be doped into the films in larger concentrations, without getting PL quenching. It should be noted that the typical concentrations used with dyes are around 1 wt%. Moreover, in some particular molecular semiconducting materials, stimulated emission was observed even in the form of neat films (non-diluted in an inert matrix). Examples of such materials are various thiophene-*S,S*-dioxide oligomers [16], spiro-type molecules [17] and the hole-transporting *N,N´*-bis(3-methylphenyl)-*N,N´*-diphenylbenzidine system (TPD) [12,19,20,21,22] (Figure 1).

Since film quality and supramolecular organization play a major role in obtaining high PL efficiencies and stimulated emission in the solid state, in the last years our group has performed detailed investigations of the ASE performance as a function of the concentration of the active molecule in the film for various types of materials [13,14,15] as well as for TPD [22]. This way, the material characteristics that lead to the best performance (lower ASE threshold, narrower ASE bandwidth and larger tunneability of the ASE wavelength) can be determined prior to the introduction of the resonant laser cavity. This kind of studies is particularly important in the case of molecular materials, since above a certain concentration the PL, and hence the stimulated emission, generally quench due to molecular interactions.

When building a laser, many different types of resonators can been used: active media between two mirrors, vertical microcavities [4,6], microrings, microdisks, etc [4,6]. An alternative way of getting feedback consists in incorporating a periodic modulation of the refractive index by a surface-relief grating (on the substrate or on the active medium) in order to obtain light reflected by diffraction or “Bragg-scattering” [4,6,23,24]. In this type of devices, called distributed feedback (DFB) lasers, light propagating in a waveguide mode is scattered by the periodic corrugation, so the scattered light from each corrugation combines coherently and an new “Bragg-scattered” wave is formed propagating in a new direction. Lasing occurs close to the Bragg wavelength (*λ*_Bragg_) given by the Bragg condition [23]:

*mλ*_Bragg_ = 2 *n_eff_Λ* (2)

where *n*_eff_ is the effective refractive index of the waveguide, *Λ* the period of the grating and *m* the order of diffraction. This lasing wavelength can be tuned by varying *n*_eff_ (by changing the refractive index or the film thickness) or *Λ*. For first-order diffraction (*m* = 1) light is diffracted out to the edge of the film, while for the case of second-order diffraction (*m* = 2) light is diffracted out perpendicular to the plane of the waveguide.

Most of the activity in recent years in the field of semiconducting organic solid-state lasers has focussed in DFB structures [6]. Two- and three-dimensional gratings have been used, providing more efficient feedback, thus improving the laser thresholds and the directionality. Several methods have been used to record the gratings: holographic methods, electron-beam lithography and soft lithography. The capability of controlling the emission characteristics of TPD by the knowledge provided by our detailed ASE studies of this material [12,20,21,22], together with the fact that TPD is one of the molecular materials with lowest ASE thresholds prompted us to build up optically pumped second-order DFB lasers using TPD as active laser material.

In this review we aim to compile the work done with TPD in view of its use as active material in optically-pumped solid-state lasers. Once it is shown that the mechanism responsible for the observation of gain is ASE, the influence of several material parameters such as film thickness, TPD concentration and waveguide characteristics, is investigated. These detailed studies were useful to determine the material parameters that provide the best ASE performance. Finally, the influence in the emission properties of the inclusion of a second-order distributed feedback in such high-performing materials is also discussed.

## 2. Results and Discussion

### 2.1. Study of the presence of stimulated emission and identification of the responsible mechanism

**Figure 2:**
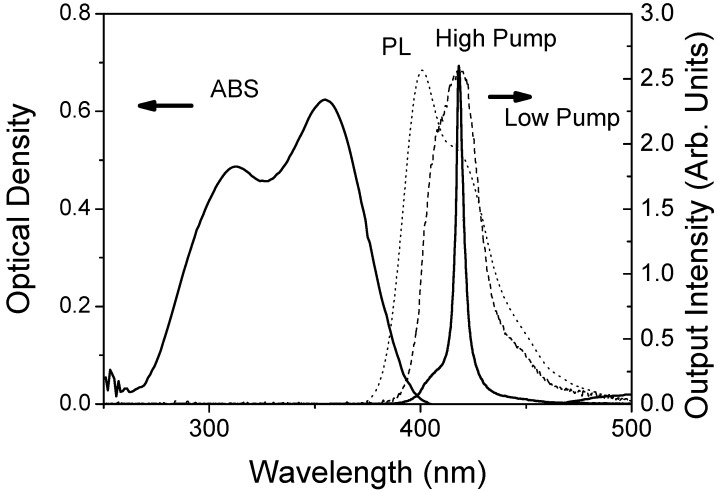
Emission spectra and optical absorption of a 20 wt% TPD-doped PS film: Optical density (ABS, full line, left axis), photoluminescence spectrum recorded in a fluorimeter (PL, dotted line, right axis), emission spectra recorded in the setup for the identification of stimulated emission at low pump intensity (0.7 kW/cm^2^; dashed line, right axis) and high pump intensity (7.5 kW/cm^2^; solid line, right axis). The high pump intensity emission spectrum has been divided by 44 for comparison purposes.

Emission spectra of PS films doped with TPD were obtained by pumping them at 355 nm as described in the Experimental section. The presence of stimulated emission was evidenced by the observation of a collapse of the width of the emission spectrum whenever the pump intensity was above a well defined value identified as the threshold. As an illustration, Figure 2 shows the emission spectra of a 20 wt% TPD-doped PS film, obtained at low and high pump intensities with a constant pump stripe length of 3.5 mm.

Its absorption and PL spectra, measured in a spectrophotometer and a fluorimeter respectively, have also been included. The PL spectrum consists of two main peaks: a main band (0-0 transition) with a maximum at around 400 nm and a first vibronic peak (0-1 transition) at around 420 nm. As observed, gain narrowing takes place at the wavelength of the first vibronic peak, as contrary to diluted liquid solutions, for which this occurs at the wavelength of the 0-0 transition [22]. This can be explained in terms of losses. In the films, due to the spectral overlap between absorption and emission at shorter wavelengths, losses are larger at the blue end of the emission spectrum. In consequence, net gain at the 0-1 transition is larger than in the main band. It should also be noted that the shape of the spectrum obtained at low pump intensity in the setup for the identification of stimulated emission, differs from that of the PL spectrum recorded in the fluorimeter. The former one is dominated by the 0-1 transition, while the 0-0 transition practically is not observed. In contrast, in the PL spectrum obtained in the fluorimeter the dominant band is the 0-0 transition. Theses variations in the shape of the emission spectra are due to different experimental configuration used (see experimental) [22]. In the setup for the identification of stimulated emission, emitted light travels along the waveguide, being amplified in its way until is collected from the edge. On the other hand, in the fluorimeter, the collected light has not been guided, so it travels a much shorter distance across the film.

**Figure 3:**
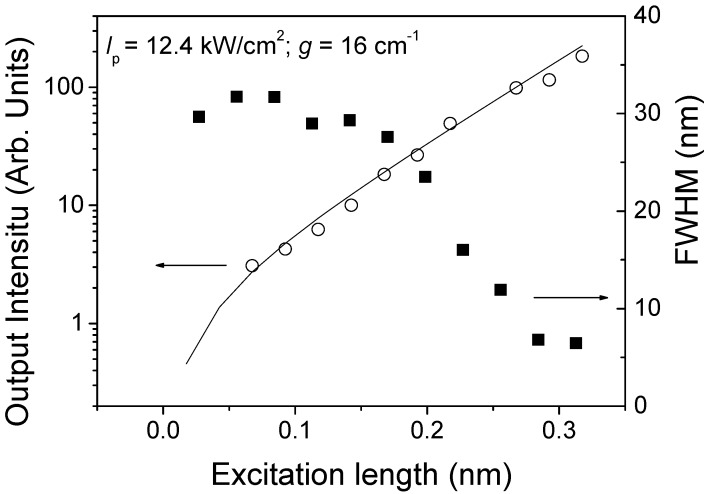
FWHM (right axis, solid square) and emission intensity at *λ* = 416 nm (left axis, open circles) as a function of excitation length, at a pump intensity of 12.4 kW/cm^2^ for a 10 wt% TPD-doped PS film. The solid line is the fit to data using Equation (1).

In order to identify the mechanism responsible for the observed collapse of the emission spectrum, we studied the dependence of the emission spectrum with the length of the pump stripe. As shown by data displayed in the right axis of Figure 3, the spectrum is broad for short pump stripes and becomes narrower when the pump stripe gets larger. This indicates that the responsible mechanism is ASE. In addition, the dependence of the output intensity at the peak of the emission spectrum (*λ* = 420 nm) on the pump stripe length (Figure 3, left axis) can be adequately fitted to Equation (1), thus providing additional support for the assignment of ASE as the mechanism accounting for the observation of gain in these systems. Moreover, from the fit, the parameters *AI*_p_ = 22.5 arbitraty units and the net gain coefficient *g* = 16 cm^-1^ were obtained. Experiments were also performed with other pump intensities. As expected, gain coefficients became larger when the pump intensity increased. In conclusion, these results indicate that ASE is the responsible mechanism for the observed spectral narrowing and rule out the presence of other phenomena, such as superfluorescence or spontaneous emission from biexcitonic state.

### 2.2. ASE performance: Concentration dependence

The dependence of ASE on the concentration of TPD in the films was investigated for a wide range of TPD concentrations, varying between 2.5 and 100 wt%. Films of similar thickness (around 425 nm) were prepared. Similarly to the 20 wt% TPD-doped film (Figure 2), a collapse of the spectrum due to ASE at sufficiently high pump intensity was observed for all the films studied, even for non-diluted films (100 wt% TPD). Nevertheless, for the neat films the quality was not very good and films degraded quickly after preparation, so reproducibility was a main problem in order to compare with the rest of the films.

**Figure 4:**
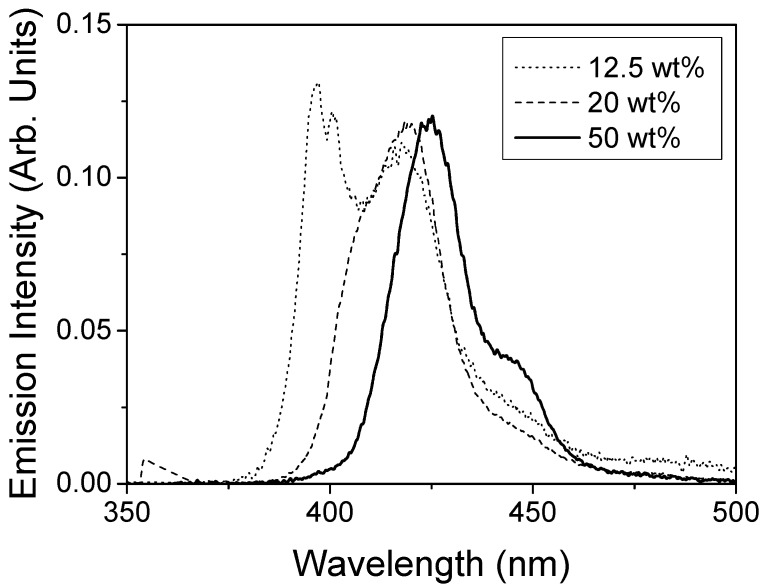
Emission spectra obtained in the setup for the identification of stimulated emission at low pump intensity (below the ASE threshold) for PS films doped with various TPD concentrations.

With respect to the emission spectra obtained at low pump intensity in the setup for the identification of stimulated emission (see experimental section), we observed that their shape was dependent of the concentration of TPD (see Figure 4). For low TPD concentrations it was similar to that of the PL spectrum obtained in the fluorimeter. On the other hand, when the TPD concentration was increased, the 0-1 transition got relatively larger, until it became dominant at high concentrations. This behavior is due to the fact that in the setup for characterizing the presence of stimulated emission, light is recorder from the edge of the film, at the end of the pump stripe. Since gain takes place at the wavelength of the 0-1 transition, this is enhanced with respect to the main band, even below the threshold. Obviously, the larger TPD concentration and the larger gain, justifying the fact that these changes are more evident in highly doped films.

The concentration dependence of ASE has been studied through several parameters: the pump intensity threshold (*I*_th-ASE_), the linewidth (defined as the full width at half of the maximum, FWHM) above threshold (FWHM_ASE_), the emission wavelength (*λ*_ASE_) and the photostability.

**Figure 5:**
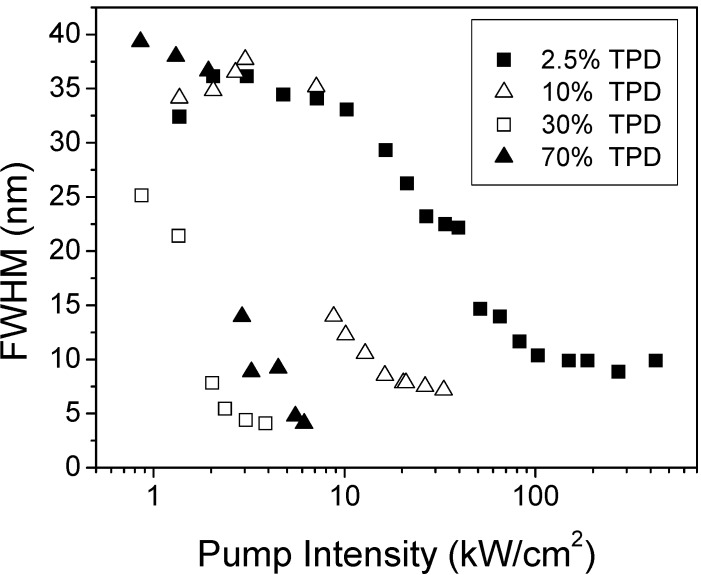
FWHM as a function of pump intensity for PS films doped with various TPD concentrations.

ASE thresholds were calculated for each sample as the intensity at which the FWHM of the emission spectrum decreases at half of its maximum value (see Figure 5). As observed, for low concentrations (*i.e.,* 2.5 wt%) the threshold was around 30 kW/cm^2^. A one-order of magnitude decrease was obtained when the TPD concentration was increased up to 20-30 wt%. For larger concentrations similar *I*_th-ASE_ values were obtained. The concentration dependence of the FWHM_ASE_ also shows this type of behavior. Indeed, the FWHM_ASE_ decreased from 9 to 5 nm when the TPD concentration was varied from 2.5 wt% up to 20-30 wt%. Then, it kept constant for larger concentrations. This type of dependence can be explained in terms of the PL efficiency of the films. We observed that the normalized emission intensity (PL integrated area divided by film thickness) increased up to around 20 wt% and then saturated and kept constant with concentration, while absorption increased linearly with concentration. Presumably, this saturation of the emission intensity is due to molecular interaction, that seems to be more important for TPD concentrations above 20 wt% [22]. It is somewhat surprising the fact that, in spite of the saturation effects observed for concentrations above 20 wt%, ASE is still observed. Moreover, thanks to possibility of adding such large amounts of active material without getting ASE quenching, ASE thresholds are much lower than those obtained with traditional dyes. Note that typical concentrations for lasing with traditional dyes are around 1 wt%.

Another striking observation for TPD-based films, is the fact that the shape of both the absorption and the PL spectra practically do not change with concentration [25], although as already mentioned, some kind of interaction mechanism seems to be taking place at concentrations above 20 wt%. Aiming to understand such behavior we decided to perform a detailed analysis based on density functional theory methods of the absorption, PL and Raman data of TPD, both in solution and in the form of thin films [25]. TPD has a twisted shape in the ground state and becomes almost planar in the excited state. In addition, results have shown that the influence of molecular interactions is much smaller than that of the torsional modes of TPD at very low frequency. Thus its photophysics can be understood from the properties of the molecule itself, even for highly-doped films.

**Figure 6:**
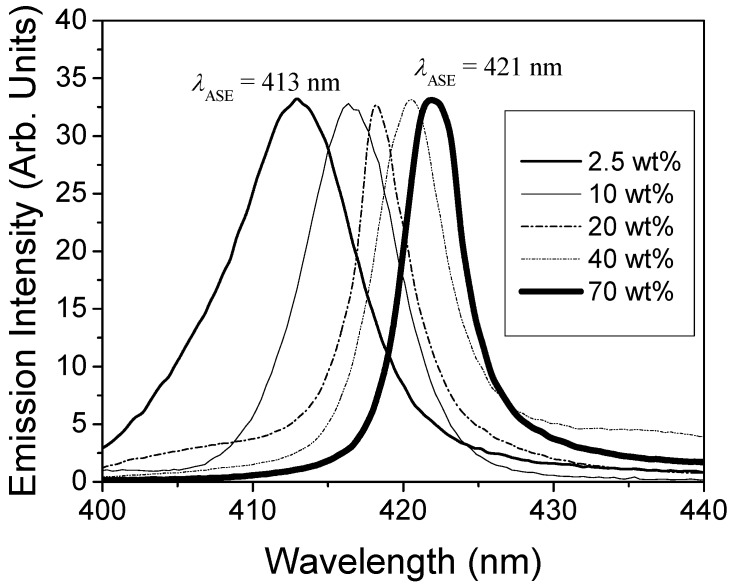
ASE spectra for PS films doped with various TPD concentrations.

The concentration dependence of the ASE emission wavelength was also investigated. As observed in Figure 6, *λ*_ASE_ could be tuned between 413 and 421 nm by changing the concentration of TPD. It is important to note that when the TPD concentration increases, the overlap between absorption an emission also increases, and the polarity of the medium also grows, so the emission would tend to shift to longer wavelengths. These shifts are also observed in the PL emission [22]. So, theses shifts are due to the increase of absorption and polarity of the medium with the concentration.

Finally, the photostability of the films was studied for several TPD concentrations. The total ASE intensity emitted under a constant pump intensity just above the threshold, was recorded as a function of time. The presence of photodegradation was evidenced by the observation of a decrease in the total ASE output, reaching half of its maximum value at around 2.5-3 min (*i.e.,* 1500-1750 pulses). After 10-12 min the emission spectra get broad, indicating that ASE does no longer exit. It was also observed that the photodegradation was highly dependent on the pump intensity [22].

### 2.3. ASE performance: Thickness dependence

The dependence of ASE, in terms of threshold, final linewidth and emission wavelength, on film thickness, was investigated in PS films doped with 15 wt% of TPD and thickness varying between 80 and 1,000 nm. In addition, the waveguiding properties of the films and their influence in the ASE performance were also studied.

**Figure 7:**
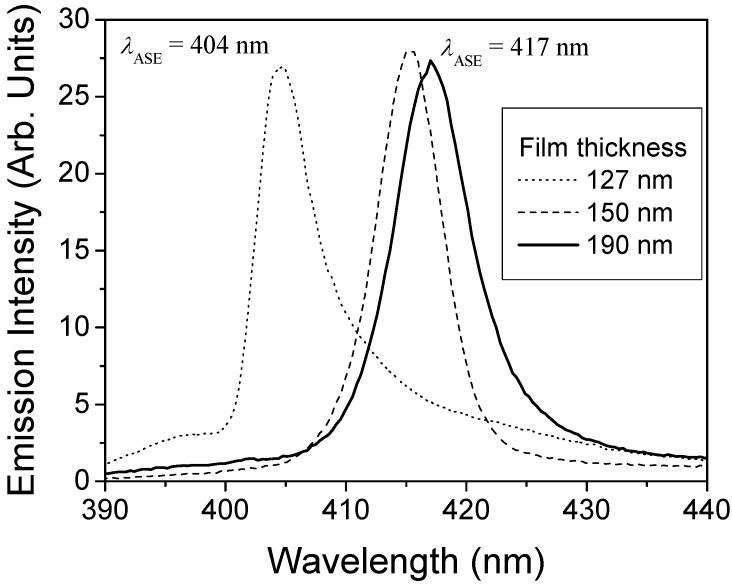
ASE spectra for 15 wt% TPD-doped PS films of various thickness.

Emission spectra of the films were obtained under optical pump at 355 nm in the ASE setup described in the experimental section. The collapse of the emission spectra due to ASE, at pump intensities above the threshold, reaching FWHM values of around 5-7 nm, was observed for all films with thickness larger than 105 nm. Figure 7 represents the ASE spectra for films of different thickness. For thickness larger than 200 nm the emission peak remains constant at a wavelength of around 417 nm. On the other hand, for thickness between 105 and 200 nm *λ*_ASE_ shifts from 404 to 417 nm.

In order to understand these results the waveguiding properties of the films were both measured and modelled. The experimental characterization was performed by the m-line technique at 633 nm. For thickness larger than 220 nm, two propagation modes were observed (one TE and one TM) and their effective indexes determined. In addition, the refractive index of the film at 633 nm was obtained through Equation (3), getting the same value (n = 1.61) for both, the TE and the TM mode, indicating that films are isotropic. In order to obtain the refractive index at the ASE emission wavelength, we assumed that the refractive index is proportional to the concentration of each component in the film. The cut-off thickness at each ASE wavelength was calculated from Equation (5) by taking into account the refractive indexes previously calculated at the corresponding ASE wavelength. This cut-off thickness determines the minimum thickness required for the propagation of one mode. In our case, the cut-off thickness at the wavelength of 417 nm is 110 nm, in agreement with the minimum thickness below which ASE was not observed (105 nm). It should be noted that for films with thickness very close to this value (107 and 127 nm) ASE is forced to take place at a lower wavelength (λ = 404 nm) in order to satisfy Equation (5).

However, the cut-off condition does not explain the variations observed in the ASE wavelength for thickness between 127 and 190 nm. In these cases the shifts observed were explain in terms of the shape of the emission spectrum at low intensities [21].

**Figure 8:**
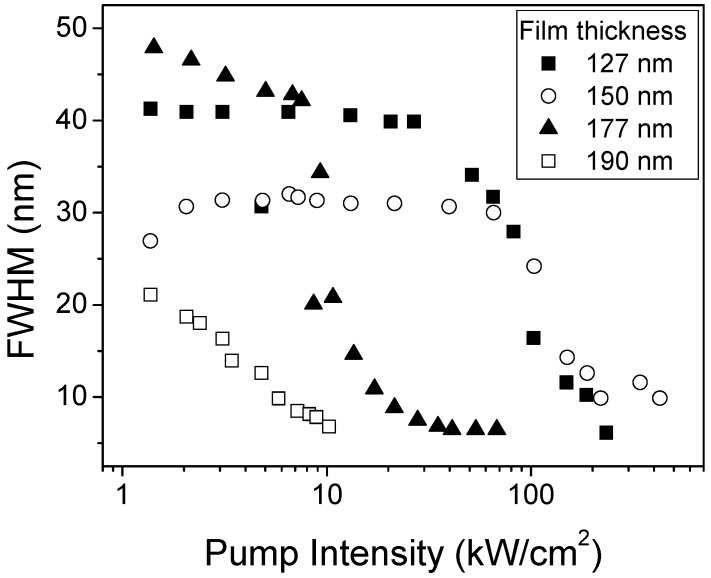
FWHM as a function of pump intensity for 15 wt% TPD-doped PS films of various thickness.

Concerning the ASE thresholds (see Figure 8), the thresholds are large for very thin films, then they decrease when the thickness increases reaching a minimum value for thickness around 200 nm. For thicker films, thresholds keep approximately constant.

This behavior has been interpreted in terms of the different confinement of the waveguide modes. This parameter is proportional to the inverse of the penetration depth into the substrate and the cover (*x*_total_), given by Equation (8). One would expect that for bad confined modes (large *x*_total_ values), the threshold would be larger, while for well confined modes (small *x*_total_ values) the threshold would decrease. Data displayed in Table 1 show that there is a good correlation between the behaviors of thresholds and mode confinement as a function of film thickness. This is a indication that the observed decrease in the ASE thresholds when the film thickness increases is due to the fact that the propagation modes are better confined.

**Table 1:** ASE thresholds and penetration depth of the waveguide mode (*x*_total_) for TPD-doped PS films of various thicknesses.

*h*^a^	*X*_T_^ b^	*I*_th-ASE_^ c^
107	1116	129
137	482	103
150	315	112
160	273	9
190	273	7
240	223	7

^a^ *h*: Film thickness (nm); ^b^*X*_total_: Total penetration depth (nm); ^c^*I*_th-ASE_: Pump intensity thresholds for ASE observation (kW/cm^2^).

### 2.4. Comparison of ASE performance of various materials

In order to adequately compare the potential of different types of materials for laser purposes, ASE thresholds rather than laser thresholds should be considered. If laser thresholds are compared, it is not clear whether the differences in thresholds are due to de material or to the cavity.

TPD presents ASE thresholds [22] as low as 1 kW/cm^2^ for PS doped with 30 wt% of TPD, that are among the lowest reported in the literature for molecular materials. Moreover, they are comparable to those of typical semiconducting polymers [17,22,26,27,28,29,30,31,32]. It is important to note, that in the case of PS films doped with TPD the lowest thresholds were obtained with films doped with 20-30 wt% of active material, while in the case of polymers they are neat films (100% of active material). Note that for comparison purpose, the thresholds must be given in power density units (W/cm^2^). These units are the appropriate ones in order to properly compare the performance of different materials, as described in detail in reference [15].

### 2.5. Laser emission in a second order DFB laser based on films doped with TPD

In order to evaluate the effect of the cavity in the emission spectrum of our material, we studied second-order DFB lasers based on PS films doped with 30 wt% of TPD. In a second-order DFB laser, optical feedback is provided through second-order Bragg scattering of the waveguided light. So taking into account the Bragg condition (Equation 2 with *m* = 2) and typical values of the effective refractive index (*N*_eff_ ~ 1.554) and emission wavelength (*λ* ~ 420 nm) of 30 wt% TPD-doped films, the grating period *Λ* was estimated as approximately 270 nm. Thus, surface relief gratings with a period of *Λ* = 270 nm (± 2 nm) and a depth of 70 nm (± 15 nm) were recorded in glass substrates by holographic lithography and subsequent RIBE methods (see Experimental). PS films containing 30 wt% of TPD were then spin-coated on top of the gratings to form active waveguides with thickness varying between 150 and 300 nm.

**Figure 9:**
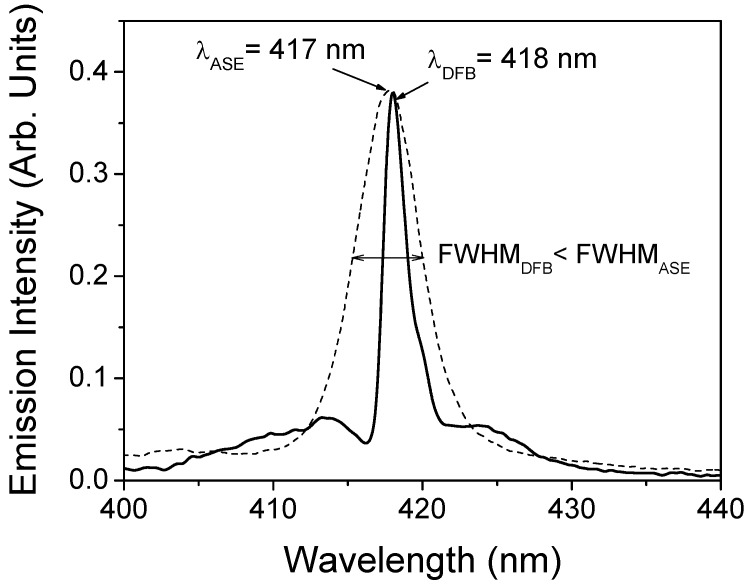
DFB emission of a TPD-doped PS film deposited over glass with a DFB grating (full line) and ASE emission of a similar film over a bare glass substrate without grating (dashed line).

The laser properties of these devices were characterized in the experimental setup described in the Experimental section, that differs in some aspects from that used in previous sections. In order to study the effect of the grating in the emission spectrum, results were compared to those obtained in the same setup for films deposited over bare glass substrates. Figure 9 shows the emission spectra at high pump intensity for a DFB device based on a 250 nm-thick polymer film and for a sample consisting in a similar film but deposited over bare glass. As observed, the presence of the grating is evidenced in the changes observed in the emission spectra of the DFB device due to waveguided PL which has been Bragg-scattered out of the waveguide at an angle normal to the substrate. This particular device emits at around 418 nm with a linewidth lower than 2 nm limited by the resolution of our spectrometer, just at the high-wavelength side of a characteristic dip. This Bragg dip is a photonic stop band for waveguided modes and is due to the inhibition of the propagation of the waveguided light by the grating [33]. The fact that lasing occurs at the edge of the dip is an indication that index modulation, rather than gain modulation, is the physical mechanism accounting for the presence of gain [2,24,33,34]. In the case of the sample without grating, a collapse of the emission spectrum is observed, in this case due to the presence of ASE. In this case, the ASE linewidth is around 5 nm, larger than that measured with the DFB structure. In addition, the ASE threshold obtained for the film without grating, was two times larger than the one obtained with the DFB device.

Finally, the possibility of tuning the DFB emission wavelength over the TPD emission spectrum was also studied. The emission wavelength of the DFB device could be tuned between λ = 415 and λ = 427 nm by varying the film thickness of the active film between 160 and 298 nm. The lowest thresholds were obtained for the devices emitting at values close to the vibrational peak of the PL spectrum of TPD (415 and 418 nm). For devices emitting at longer wavelengths, the thresholds increase and peaks due to ASE are also observed.

In summary, the presence of DFB gratings in TPD-doped PS films has lead to reductions of the laser thresholds in 50% and in the emission linewidth, from 5 nm (without grating) to less than 2 nm. In addition, the emission wavelength of the structures was tuned in the range 415-427 nm by changing the thickness of the active film.

## 3. Experimental Section

### 3.1. Sample preparation

Organic films were prepared by spin-coating a solution containing PS as an inert polymer doped with TPD over glass substrates (BK7). The concentration of TPD was varied between 5 and 90 wt%, while the concentration of the solid materials with respect to the solvent (toluene) was adjusted to get films of similar thickness. In the thickness dependence studies, a fixed concentration of TPD was used (15 wt%) while the thickness was varied by changing the concentration of PS, with respect to the solvent. TPD neat films (*i.e.,* 100 wt% of TPD and no PS) of thickness 150 nm also were prepared. Films thickness were measured by means of an interferometer coupled to an optical microscope Film quality (transparency and homogeneity) was good for concentrations of TPD up to 70 wt%, but for larger concentration films remained transparent just for a few hours after preparation. DFB devices were prepared by using corrugated glass substrates. Surface relief gratings were engraved on substrates by a holographic method [35] follow by conventional reaction ion beam etching.

### 3.2. Optical spectroscopic characterization

**Figure 10:**
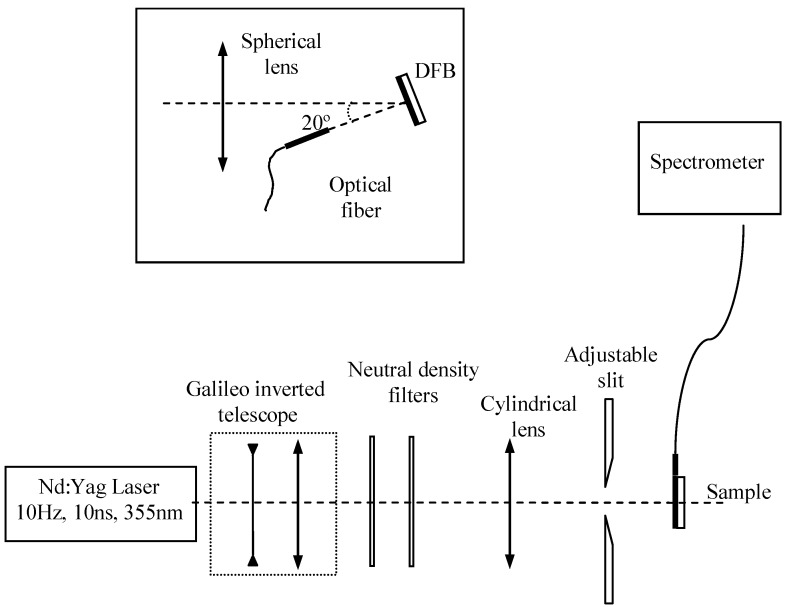
Experimental setup for characterizing the presence of stimulated emission in waveguide configuration. Inset: Detail of the pump and recording configuration in the case of DFB lasers.

Absorption and PL spectra were obtained using a Shimadzu spectrophotometer and a Jasco FP-6500/6600 fluorimeter, respectively. In the fluorimeter, samples were excited at 355 nm and the beam was collected at a 45º angle to avoid the pump beam. The experimental setup used to investigate the presence of stimulated emission in waveguide configuration is shown in Figure 10 [11].

Samples were photopumped at normal incidence with a pulsed Nd:YAG laser (10 ns, 10 Hz) operating at 355 nm, which lies in the absorption region of TPD. The energy of the pulses was controlled using neutral density filters. The laser beam was expanded and collimated. A cylindrical lens and an adjustable slit were then used to shape the central part of the beam into a stripe with a width of approximately 0.53 mm and a length that could be varied between 0.1 and 3.5 mm. The pump stripe was projected right up to the edge of the film where the emitted light was collected with a fiber spectrometer. In the characterization of DFB lasers, the cylindrical lens was substituted by a spherical lens that focused the central part of the pump beam in a spot of 1,3 mm diameter on the sample (inset of figure 10). The pump beam was incident at 20º and the emitted laser beam, which was output coupled by second-order Bragg scattering, was collected in a direction perpendicular to the film with the fiber spectrometer placed at 1 cm of the sample.

### 3.3. Waveguide characterization and modelling

The waveguiding properties of the films were characterized by the m-line technique at 633 nm provided by a He-Ne laser. The films under study constitute asymmetric planar waveguides, since their refractive index is larger than those of the substrate (glass, s) and the cover (air, c). The equation for the guided modes is given by [36]:

*k n_f_ h cosθ_m_ + ϕ_s_ + ϕ_c_ = m π* (3)

where *k* is the free space wave vector, *n_f_* and *h* are respectively the refractive index and the thickness of the organic film, *m* is an integer that accounts for the mode order, *θ_m_* is the angle of propagation for mode of *m*-order and *ϕ_s_* and *ϕ_c_* are half of the phase changes at the interfaces film-substrate and film-air, respectively. The equations for *ϕ_s_* and *ϕ_c_* for TE and TM polarizations read:

(4) $$\tan\phi_{c,s}^{TE}=-\sqrt{N_{eff}^{2}-n_{c,s}^{2}}/(n_{f}\cos\vartheta )\;\mathrm{and}\;\tan\phi_{c,s}^{TM}=(n_{f}^{2}/n_{c,s}^{2})\tan\phi_{c,s}^{TE}$$

where *N_eff_ = n_f_ sinθ* is the so-called effective index.

The procedure we generally use consists in experimentally determining the effective indexes for the different modes of a certain waveguide by the m-line technique. Then, by solving Equation (3) numerically, the refractive index and the thickness of the film can be determined. As described in next section, all the waveguides studied here had just one propagation mode (for each polarization), so Equation (3) was used to determine the refractive index of the film by using the film thickness values measured by interferometry.

It can be shown that for *h/λ* values below a certain quantity there is no solution for Equation (3). In other words, for a certain mode, there is a minimum thickness (at a given wavelength) below which the mode can not propagate. Similarly, for a given thickness, the mode can not propagate for wavelengths above the so-called cut-off wavelength. The cut-off condition for the zero-order mode at TE polarization is given by [36]:

(5) $$(h/\lambda )=\tan^{-1}\sqrt{\left(n_{s}^{2}-n_{c}^{2})/(n_{f}^{2}-n_{s}^{2}\right)}/\left(2\pi \sqrt{n_{f}^{2}-n_{s}^{2}}\right)$$

In order to evaluate the confinement of the different propagation modes one should take into account that the light penetrates in the substrate and the cover. These penetration depths (*x_s,c_*) can be expressed as [36]:

(6) $$x_{s,c}=z_{s,c}/\tan\vartheta$$

where z_s,c_ values for TE and TM polarizations are given by:

(7) $$z_{s,c}^{TE}=\tan\vartheta /\left(k\sqrt{N_{eff}^{2}-n_{s,c}^{2}}\right)\;\mathrm{and}\;z_{s,c}^{TM}=z_{s,c}^{TE}/\left(\frac{N_{eff}^{2}}{n_{s,c}^{2}}+\frac{N_{eff}^{2}}{n_{f}^{2}}-1\right)$$

The larger the penetration depth, less confined the mode becomes. Therefore, the lack of confinement of the mode is directly related to the total penetration depth into the substrate and the cover (air in this case):

(8) $$x_{total}=x_{s}+x_{c}$$

## 4. Conclusions

The laser properties of polymer films based on the hole-transporting organic molecule *N,N´*-bis(3-methylphenyl)-*N,N´*-diphenylbenzidine system (TPD) have been investigated. Detailed studies of the dependence of their amplified spontaneous emission on various material parameters such as film thickness and TPD concentration have allowed to optimize their laser performance. In particular, the lowest thresholds (1 kW/cm^2^) and linewidths (**~**5 nm) were obtained for TPD concentrations of around 20-30 wt% and thickness well above the cut-off thickness for the propagation of one mode. These ASE thresholds are among the lowest reported to date for molecular materials, no energy transfer processes being present. In addition, a further improvement in the laser performance was obtained by including a DFB grating in the substrate, obtaining linewidths <2 nm and laser thresholds 50% lower than the ASE thresholds observed in films without gratings. The emission wavelength of the DFB structures has been tuned in the range 415-427 nm by changing the thickness of the active film.
